# Bioinspired Control of Calcium Phosphate Liesegang Patterns Using Anionic Polyelectrolytes

**DOI:** 10.1021/acs.langmuir.1c02980

**Published:** 2022-02-11

**Authors:** Young Shin Cho, Miyoung Moon, Gábor Holló, István Lagzi, Sung Ho Yang

**Affiliations:** † Department of Chemistry Education, Korea National University of Education (KNUE), Chungbuk 28173, Republic of Korea; ‡ MTA-BME Condensed Matter Physics Research Group, Budapest University of Technology and Economics, Budapest H-1111, Hungary; § Department of Physics, Budapest University of Technology and Economics, Budapest H-1111, Hungary

## Abstract

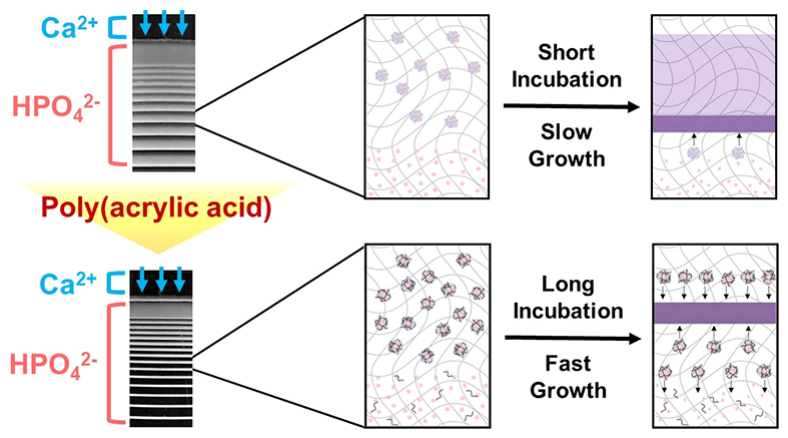

The Liesegang phenomenon is a spontaneous pattern formation, which is a periodic distribution of the precipitate discovered in diffusion-limited systems. Over the past century, it has been experimentally attempted to control the periodicity of patterns and structures of precipitates by varying the concentration of the hydrogel or electrolytes, adding organic or inorganic impurities, and applying an electric or pH field. In this work, the periodic patterns of calcium phosphate were manipulated with an anionic macromolecular additive inspired by bone mineralization in which various noncollagenous proteins are involved in the formation of a polymer-induced liquid precursor. The periodic patterns were systematically controlled by adjusting the amount of poly(acrylic acid), and they were numerically simulated by adjusting the threshold concentration of nucleation. The change of the pattern is explained by improved stability and directional diffusion of the intermediate.

## Introduction

The spontaneous self-organization and self-assembly of components into controllable microstructures and periodic patterns have gained growing interest in the field of material science.^1−4^ The Liesegang phenomenon is one of the spontaneous pattern formation, which is a periodic distribution of the precipitate discovered in diffusion-limited systems. Over the past century, numerical models have been developed for explaining the spatial periodicity of Liesegang patterns and for improving their accuracy, regularities, and validity in terms of macroscopic quantities such as the position of the precipitation zones, their appearance time, and widths.^5^ On the other hand, it has been experimentally attempted to control the periodicity of patterns and structures of precipitates^2,5−13^ with aims of potential applications in materials science for a bottom-up fabrication tool,^3,14^ as well as fundamental studies in biogenic and geological patterns.^15−17^ The pattern and structures have been controlled by varying the concentration of the hydrogel or electrolytes, adding organic or inorganic impurities, and applying electric or pH fields.^8,10−13,18−20^

In most previous studies, the Liesegang phenomenon has been numerically interpreted based on a simple precipitation mechanism. However, in the view of crystallization, the phenomenon could be interpreted as more complicated processes including nucleation, growth, and phase transformation.^21^ Furthermore, there is an increasing number of discoveries, suggesting that the phenomenon occurs through the attachment of amorphous intermediate particles larger than atoms/ions/molecules.^22−24^ In a recent crystallization theory, it has been emphasized to investigate characteristic properties of amorphous intermediates, such as their stability, diffusivity, or reactivity.^25−27^ In this context, the recent viewpoint of crystallization can give insight into the control and interpretation of the Liesegang phenomenon.

The field of crystallization and biomineralization has drawn great attention because organic/inorganic hybrid materials formed by a biological process have superior mechanical properties such as high stiffness, toughness, and low brittleness.^28−30^ Among the many biominerals, calcium phosphate (CaP), the main component of hard tissues of vertebrates, has been intensively studied with the purpose of biomedical treatment on physical damage or loss of bone, as well as fundamental understanding of the pathological pathway of bone disease.^31,32^ In the process of bone formation, various noncollagenous proteins (NCPs) are involved in the formation and transformation of biogenic CaP through specific interactions at the inorganic/organic interface.^33^ For example, mineralization inhibitors, including osteopontin, osteocalcin, and fetuin, stabilize amorphous calcium phosphate (ACP), which is an intermediate precursor for a mature bone.^33,34^ In the early stage of mineralization, it is generally postulated that ACP stabilized by NCPs has a liquidlike property called the polymer-induced liquid precursor (PILP).^35,36^ Because of its fluidity, NCP-stabilized ACP can infiltrate into the supramolecular structure of collagen by capillary action or electrostatic interactions. Many of the NCPs are negatively charged caused by abundant carboxylate groups in aspartic and glutamic acid residues.^37^ By mimicking the anionic nature of NCPs, anionic organic polymers such as poly-l-aspartic acid (pAsp) or poly(acrylic acid) (PAA) have been used for controlling the nucleation and growth of CaP.^36,38^ The biomimetic process has been widely used for producing integrated organic/inorganic structures by taking full advantage of polyanionic-stabilized mineral precursors including wettability, morphological changeability, and diffusible abilities.

Recently, we found the inhomogeneity in single bands of CaP Liesegang patterns formed under slow and controlled diffusion in a gelatin medium. It was demonstrated that the overall structure of each band was dependent on the diffusive properties of ACP precursors.^21^ Although anionic polymers have been used in biomimetic approaches to generate complicated microstructures of biogenic materials, it has not been used to control the Liesegang phenomenon. In a few previous reports, soluble organic molecules have been added to control polymorphs, composition, and hierarchical organization of CaCO_3_ and BaCO_3_.^39,40^ However, generated periodic patterns were formed only at the surface of a single crystal, and it was not spatially extended.^39,40^ We speculated that the periodic patterns of the Liesegang bands also can be precisely controlled by adopting the role of NCPs that stabilize the intermediates during biomineralization.

Inspired by biomineralization, here, we show, for the first time, how to control CaP Liesegang patterns with an organic macromolecular additive in contrast to the previous reports that have used single ions or molecules. PAA was used as an analog of NCPs, and CaP crystallization was performed in a gelatin hydrogel mimicking a collagen matrix. The periodic patterns were systematically controlled by the amount of PAA, and they were numerically simulated by adjusting the threshold concentration of the nucleation. The change in the pattern is explained by the improved stability and fluidity of the intermediate.

## Experimental Section

### Materials

Calcium nitrate tetrahydrate (Ca(NO_3_)_2_·4H_2_O, 99%). Sodium phosphate dibasic (Na_2_HPO_4_, 99.0%), poly(acrylic acid) (PAA, MW = 1.8 kDa, viscosity: ≤2000 cP, Tg: 106 °C), and gelatin (type A, gel strength 300) were purchased from Sigma-Aldrich, and ultrapure water (18.2 MΩ cm) from a Direct-Q system (Merck Millipore, Germany) was used.

### Settings

The hydrogel was prepared by mixing 3.5% (w/v) gelatin with a 0.05 M Na_2_HPO_4_ solution, and the mixture was heated to 65 °C until the solution become homogenous. In the experiments with PAA polymers, a solution of PAA was also added to the Na_2_HPO_4_ solution initially to give the desired concentration (0.01, 1.0, and 2.0 mg/mL) depending on the experimental condition. Then, the obtained hot gel solution was cooled at room temperature for gelation. After that, crystallization experiments were performed in a single-diffusion system in which the Ca(NO_3_)_2_·4H_2_O solution was poured into the preformed gelatin gel mixture. Subsequently, the reaction was observed at room temperature for 5 days.

### Characterization

The structure of each band was identified with an intensity profile based on the variation of lightness. The photograph was converted to an 8-bit image, and the lightness of each pixel was measured with the gray level using Image J. The spatiotemporal development of a single band was obtained from the setup for a cylindrical hydrogel, which was used in our previous work.^21^ Three-dimensional (3D) curves were recorded using a camcorder (HDR-CX360, Sony, Japan) and processed with the line profile function of MATLAB (R2017a, The Mathworks, Inc., USA). The morphology of CaP precipitates was investigated by field emission scanning electron microscopy (SEM, FEI Co., Netherlands). For SEM analysis, the gel column containing crystalized CaP was sliced into sections, and the precipitate in these slices was dehydrated in 98% ethanol solution. The dehydrated gels were critical-point-dried with carbon dioxide (CO_2_) using a critical point dryer (SAMDRI-PVT-3D, Tousimis, USA). The surface and the cutting edge of the samples were identified with an accelerating voltage of 10 kV after sputter-coating with platinum. Fourier transform infrared spectroscopy (FT-IR, Alpha eco-ATR, Bruker Optik Co., Germany) and powder X-ray diffraction (XRD, X’Pert PRO MRD, Cu Kα = 1.54 Å, PAN Analytical, Netherlands) were used to investigate crystalline phases. The samples were prepared by washing with distilled water for 1 day to thoroughly eliminate sodium chloride and then dried with a freeze dryer (TFD8503, IlShinBioBase, Korea). The X-ray tube was operated at 40 kV and 40 mA. In experiments to trace the pH variation during the band formation, a few drops of the indicator (universal indicator or bromocresol purple) were added to the mixture of 3.5% (w/v) gelatin and 0.05 M Na_2_HPO_4_ solution. In all bands, the color of the universal indicator always changed first, and the color change of bromocresol purple was followed. The hydrogel’s spatial pH was measured using a pH meter (Seven Compact, pH/Ion meter S220, Mettler Toledo, Switzerland). The diameter of the pH electrode (Mettler Toledo InLab Ultra-micro pH electrode) is 3 mm.

### Simulation

The reaction–diffusion system (a set of partial differential equations) was solved numerically using the method of lines technique: spatial discretization of the partial differential equations (eq 1) on an equidistant grid combined with a forward Euler method for the integration in time. We applied the following initial conditions *a*(*t* = 0, *x*) = 0, *b*(*t* = 0, *x*) = 1.0, *c*(*t* = 0, *x*) = 0, *p*(*t* = 0, *x*) = 0, and *d*(*t* = 0, *x*) = 0 to reflect the initial conditions in the experiments, namely, phosphate ions were uniformly distributed in the gel. We applied the Dirichlet boundary condition at *x* = 0 and no-flux boundary conditions at *x* = *L* for all chemical species, where *L* is the length of the simulation domain. At *x* = 0, we applied the following Dirichlet boundary condition for the chemical species of A: *a*(*t*, *x* = 0) = 1.0 (the concentrations of other chemical species at the boundary were zero), to reflect the fact that the calcium ions diffused from outside into the gel, keeping its concentration constant overtime at the gel interface (*x* = 0). All quantities and parameters are in dimensionless units.

## Result and Discussion

**Figure 1:**
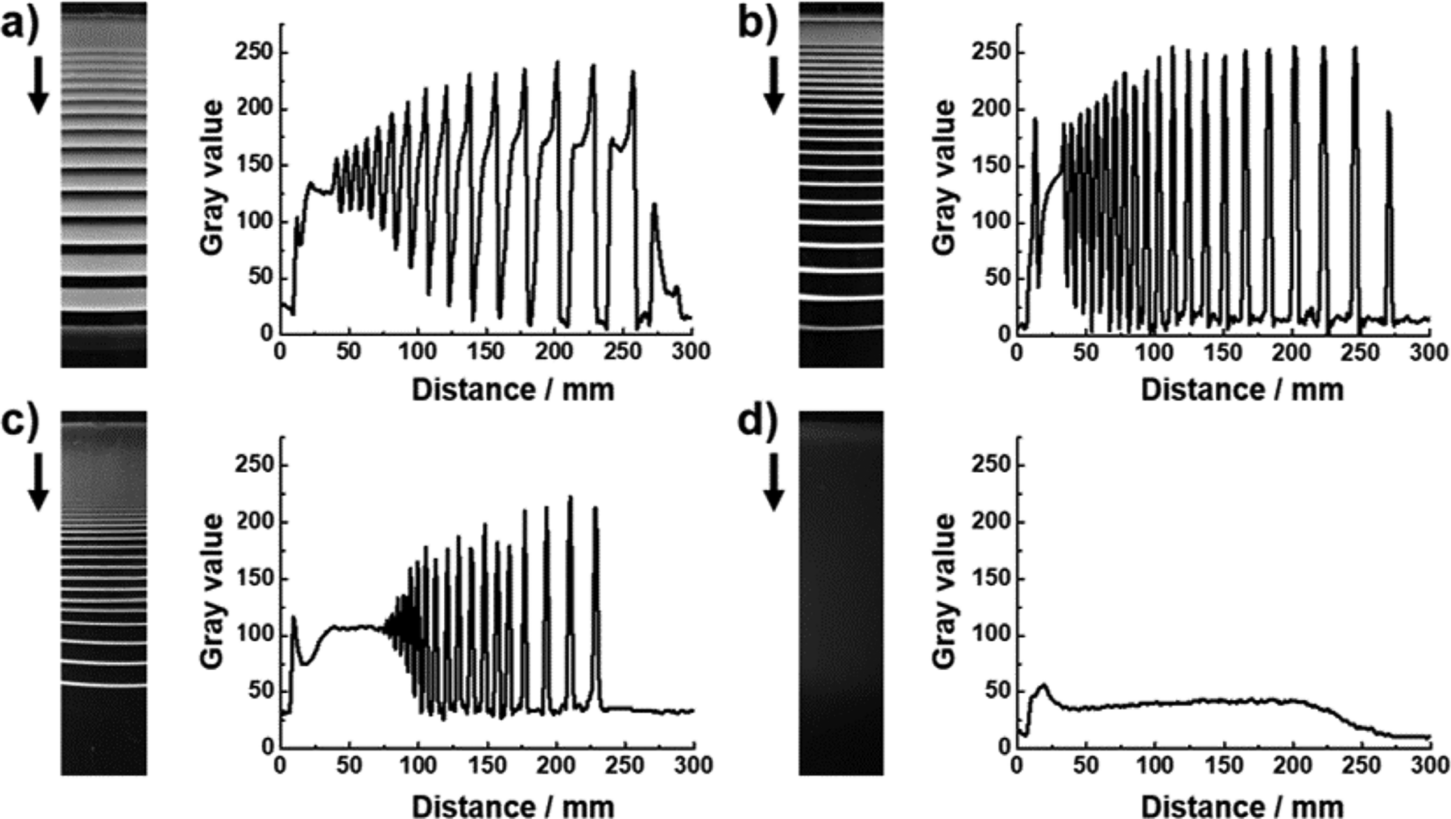
Photograph (left) and its gray value profile (right) of Liesegang patterns formed in hydrogels. The PAA concentration is (a) 0, (b) 0.01, (c) 1.0, and (d) 2.0 mg/mL. Black arrows indicate the direction of the diffusion of Ca^2+^ ions.

As Ca^2+^ diffused into gelatin hydrogel containing HPO_4_^2–^, the periodic precipitation bands of calcium phosphate (CaP) crystals were formed in the hydrogel. Four different systems were tested for investigating the influence of the macromolecular additive on the periodic bands of CaP by varying the concentration of PAA. In a control experiment, periodic precipitation was observed (Figure 1a). The pattern was confirmed as the Liesegang phenomenon by three laws: the spacing law, the time law, and the width law (Supporting Information, Figure S1). The width of the single bands was narrowed by the addition of PAA (0.01 mg/mL) and gradually decreased by increasing the amount of PAA (0.1 mg/mL) (Figure 1b, c). In a high concentration of PAA (2 mg/mL), a continuous precipitation zone was formed without the formation of periodic bands (Figure 1d). The opacity profile also confirmed the effect of the PAA additive (Figure 1). The opacity of each band was measured by the gray value obtained from the 8-bit digital images. In a control experiment, the typical pattern of Liesegang bands was observed as mentioned in our previous report.^21^ A continuous band was formed at the near reservoir, and periodic patterns consisted of asymmetric bands (Figure 1a). As the concentration of PAA increased, the opacity profile of each band became sharper, and the intensity was decreased (Figure 1b, c). In the case of 1.0 mg/mL PAA, the continuous band became wider, and the number of bands decreased. In the presence of 2.0 mg/mL PAA, the pattern disappeared, and a continuous band was formed with low intensity (Figure 1d). Because the amount of the precipitate is proportional to the opacity, the decreased opacity (caused by PAA) indicates that the density of the precipitate is reduced by the addition of PAA. The trend is consistent with the previous report that crystallization is generally inhibited by an interaction between anionic macromolecules and precursors.

**Figure 2:**
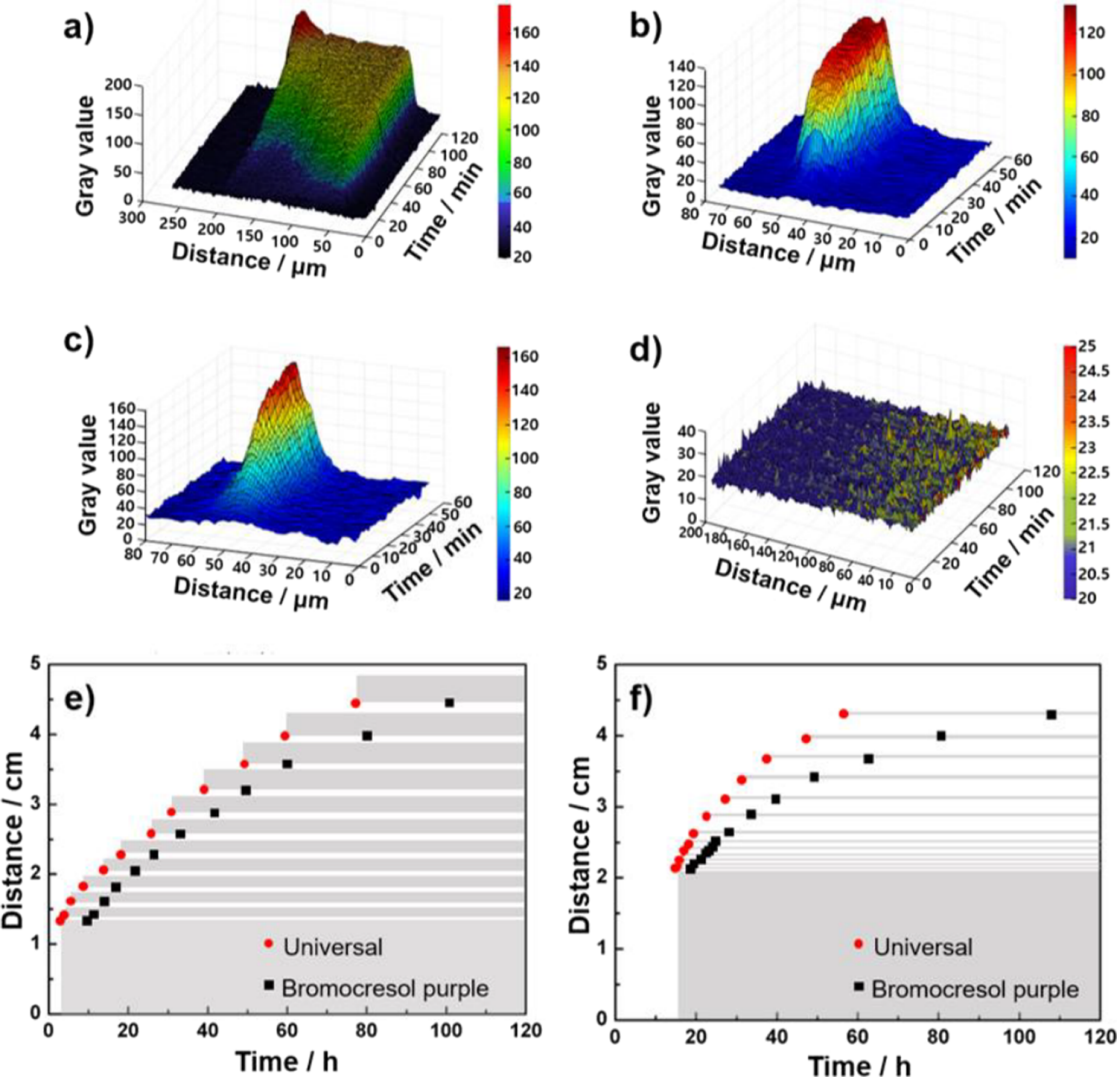
Single band formation analyzed spatiotemporally: (a) control, (b) 0.01, (c) 1.0, and (d) 2.0 mg/mL of PAA. Graph of color change measured by universal (Black Circle) and bromocresol purple (Black Square) indicators. The PAA concentration is (e) 0 and (f) 1.0 mg/mL.

The process of a single band formation in Liesegang patterns was spatiotemporally investigated (Figure 2). As the density of the precipitate can be inferred by the intensity of lightness in a hydrogel, the development of a single band was visualized with three-dimensional (3D) plots based on the variation of gray values at each location in real time. The density of CaP in every single band was assigned as different colors, depending on its numerical gray value as a function of diffusion distance and time. The lines indicated that the process of single band formation was varied, depending on the concentration of PAA. In the presence of 0.01 and 1 mg/mL PAA, a single band was formed much faster than in a control experiment, and the width of the band dramatically decreased (Figure 2a–c). In the case of 2 mg/mL of PAA, a continuous band with weak intensity was sequentially formed along the Ca^2+^ diffusion direction (Figure 2d). The band formation process could be traced by a color change of the indicator because protons are released when the Ca^2+^ ions react with HPO_4_^2–^ (Figure 2e, f). Because of the decreasing pH during crystallization, it is possible to infer the crystallization process by identifying the change in pH. Two different indicators were used to track the pH change from 8 to ∼6 with the universal indicator and from 8 to <5 with bromocresol purple. Although pH 6 and 5 did not indicate the exact point of crystal nucleation and maturation, the color change of the two indicators could be used to identify the rate of crystallization in every band formation process. When the precursors were formed at the reaction front, the pH decreased, which could be measured by the color change of the universal indicator from green to yellow. As crystallization proceeded, the pH decreased further, which could be indicated by the color change of bromocresol purple from purple to yellow. The band located farther from the Ca^2+^ reservoir had a longer time interval between the color change of the two indicators. It is noticeable that the time interval for the two indicators became longer when PAA was added in hydrogel even at nearly the same distance. For example, at ∼4.0 cm (*n* = 11, *n*th band), the color of the universal indicator changed at ∼60 h and that of bromocresol purple changed at ∼80 h in the control experiments, which means that the time interval for color change of both indicators was ∼20 h (Figure 2e). In contrast, in the presence of 1 mg/mL of PAA, the time interval for color change of both indicators was ∼33 h at the same distance (*n* = 10); the universal indicator changed its color at ∼47 h and bromocresol purple did at ∼80 h (Figure 2f). In summary, the results indicate that it takes a longer incubation time for nucleation, but the band is rapidly grown once the crystallization starts in the presence of PAA.

In fact, PAA is an acidic polymer that can influence the pH of the hydrogel, and this can directly affect the mineralization of CaP. To verify the pH change in the whole process of band formation, the spatial pH distribution of the hydrogels was measured according to the distance from the interface of the Ca^2+^ reservoir after 5 days of reaction in the presence of PAAs of 0, 0.01, 0.1, and 0.2 mg/mL, respectively (Supporting Information, Figure S2). Because protons are continuously released during crystallization, the degree of crystallization can be inferred from the pH change. The pH at ∼35 mm became lower as a function of the PAA concentration, which means that the pH of the hydrogel become acidic with the addition of PAA in the initial stage. Although the pH range varied in 6.5–7.5, the pH difference is negligible at such neutral conditions. In addition, the final pH converged into 4.3–4.5 as crystallization proceeded regardless of the concentration of PAA. The result indicated that the overall crystallization process occurred under similar pH conditions. Therefore, it was concluded that the effect of initial pH caused by the acidity of PAA was tolerable in our system.

**Figure 3:**
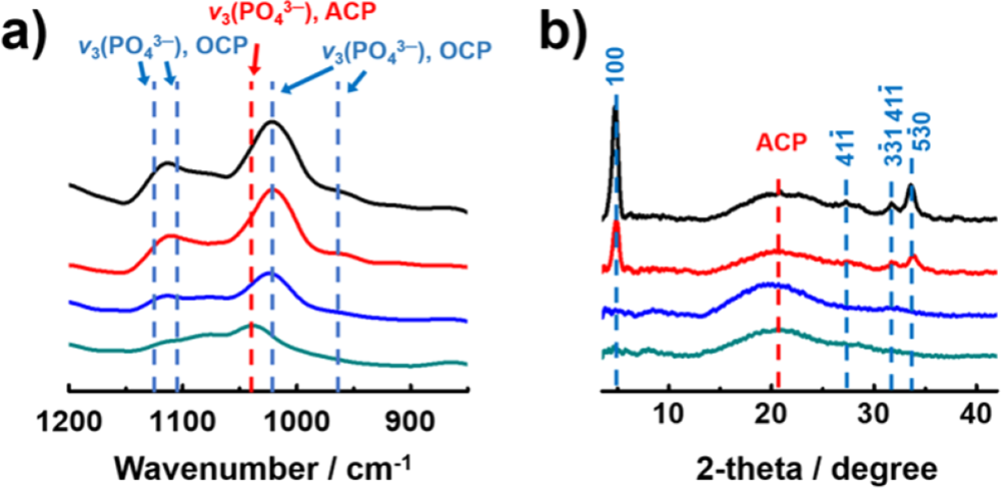
(a) FT-IR and (b) XRD of CaP formed in hydrogels. The PAA concentration is 0 (black), 0.01 (red), 1.0 (blue), and (d) 2.0 mg/mL (green).

The patterns were characterized by FT-IR and XRD (Figure 3). In the control experiment, it was confirmed that the patterns are composed of octacalcium phosphate (OCP) by the peaks at 1121, 1103, 1023, and 962 cm^–1^ in the FT-IR spectrum, which is assigned as the ν_3_ vibration of PO_4_^3–^ in OCP (Figure 3a, black line).^41^ The XRD pattern also confirmed the characteristic peaks of OCP at 4.7°, 27.4°, 31.7°, and 33.6° which are indexed as (100), (41-1), (3-11)/(41-1), and (5-30) planes in OCP crystals, respectively (Figure 3b, black line).^42^ As the concentration of PAA increased, the intensity of peaks decreased generally in FT-IR spectra. However, it is noticeable that a peak at 1040 cm^–1^ became characteristic in the spectrum of 2.0 mg/mL PAA, which is attributed to the amorphous nature of CaP (Figure 3a, green line).^43^ It indicated that the final phase of CaP was ACP under the high concentration of PAA. The amorphous nature of CaP formed with PAA was also supported by XRD analysis. The peaks originating from the OCP crystal were reduced in the spectrum of 0.01 mg/mL PAA (Figure 3b, red line) and disappeared in the spectra of both 0.1 and 1 mg/mL PAA (Figure 3b, blue and green line). Instead, a broad peak was detected at 21°, which is assigned as ACP.^44^ The results mean that the phase of CaP was changed from OCP to ACP as a function of PAA. The crystal phase of CaP formed after 1 h reaction was also investigated with XRD analysis (Supporting Information, Figure S3). In contrast to 24 h samples, the dominant phase of CaP at 1 h was ACP regardless of the concentration of PAA. This implies that ACP was commonly formed in the early stage of band formation in all experimental conditions. Considering that the peak assigned to OCP becomes less prominent as the PAA concentration increases, it is reasonable that PAA stabilizes the ACP and inhibits the transformation of ACP into OCP. It is further supported by XRD analysis on 1 h samples; the intensity of small peaks of (3-11)/(41-1) and (5-30) planes in OCP increased as the concentration of PAA decreased. Based on the results, we speculated that the variation of Liesegang patterns resulted from different stabilities of ACP during the crystallization caused by PAA.

**Figure 4:**
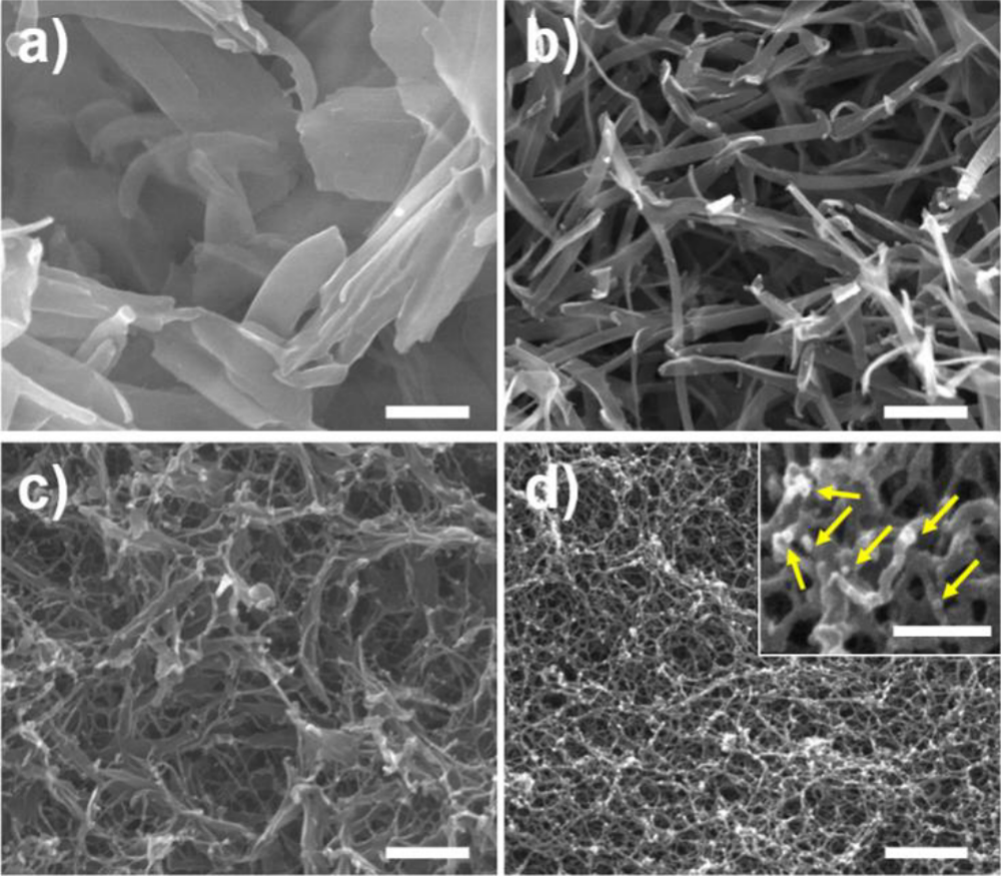
SEM micrograph of CaP formed in hydrogels. The PAA concentration is (a) 0, (b) 0.01, (c) 1.0, and (d) 2.0 mg/mL. The scale bar is 500 nm [inset in (d): 200 nm]. Yellow arrows indicate dangled CaP particles on the gelatin hydrogel.

For more detailed analysis, the microstructures of the composite were observed by SEM. In a control, platelike crystals were observed on a micrometer scale (Figure 4a). With the addition of 0.01 mg/mL PAA, the shape of crystals changed to the ribbon-like structure, which looked like a diminished structure of platelike crystals in a control (Figure 4b). In the presence of 1 mg/mL PAA, more diminished crystals were embedded in the gelatin network (Figure 4c and Supporting Information, Figure S4c). Using 2 mg/mL of PAA, only nanometer-sized granules were attached to the network (Figure 3d and Supporting Information, Figure S4d). Decreased sizes and diminished structures suggest that overall crystallization including nucleation and growth was inhibited by PAA. Considering that the platelike and spherical shape is a typical structure of OCP^21,45^ and ACP,^46^ the variation of the CaP shape indicates that the growth of OCP and transformation of ACP into OCP were more inhibited by the function of PAA that was consistent with the XRD analysis. Also, dangled precipitation on the gelatin surface at a high PAA concentration is similar to previous reports that explain the intrafibrillar mineralization of CaP induced by the addition of anionic polyelectrolytes.^35,36^

### Simulations

To describe and understand the variation of the pattern in the experiments, we modified a reaction–diffusion model, which has been used for describing asymmetric CaP Liesegang bands in our previous work.^21^ The system is described by a set of partial differential equations (PDEs) that have the form
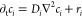
1where *c_i_*, *D_i_*, and *r_i_* are the concentrations, diffusion coefficients, and kinetic terms of the corresponding chemical species, respectively, and ∇ is the Nabla operator. The numerical solution of eq 1 describes the spatiotemporal pattern formation.

Our model comprises four independent processes. Precipitation is described with the corresponding chemical rates (*v*) and rate constants (*k*) to describe pattern formation:
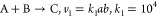
2
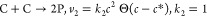
3
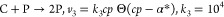
4
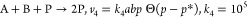
5
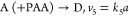
6
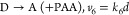
7where *a*, *b*, *c*, *p*, and *d* are the concentrations of the chemical species A (Ca^2+^), B (HPO_4_^2–^), C (intermediate), P (precipitate), and D (Ca^2+^-PAA complex), respectively, Θ is the Heaviside step function, and c*, α*, and p* are the threshold concentrations of precipitation processes described by eqs 3−5, respectively. We used chemical equations (eqs 3−5) in a form having 2P as a product to reflect the mass conservation.

**Figure 5:**
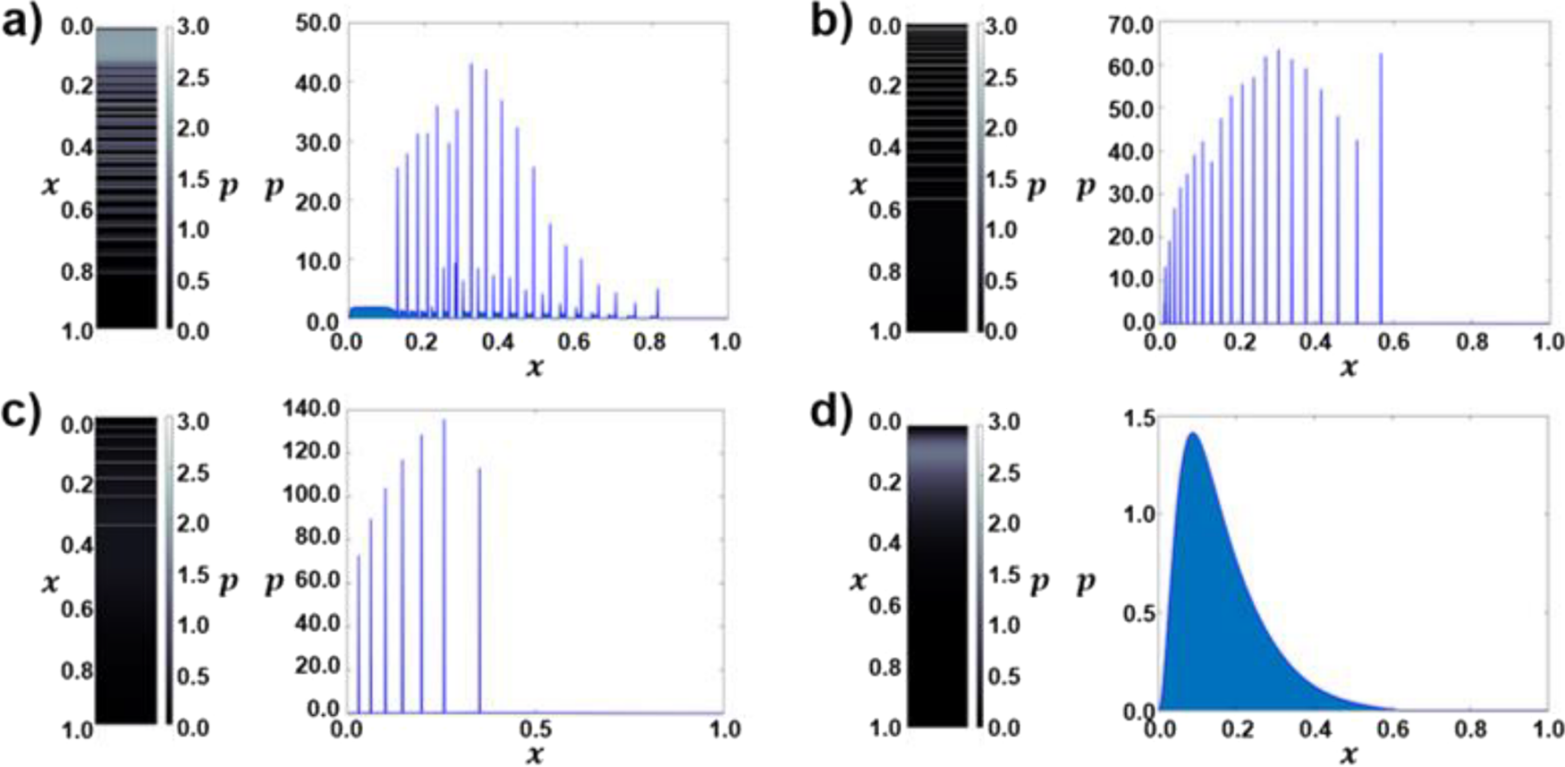
Simulated Liesegang pattern at *t* = 10. Distribution (right) and cross-sectional profile (left) of precipitates in the hydrogel when the threshold in eq 4 and reaction rate constant in eq 7 are (a) α* = 0, *k*_5_ = 0, and *k*_6_ = 0, (b) α* = 0.01, *k*_5_ = 10^2^, and *k*_6_ = 10^3^, (c) α* = 0.1, *k*_5_ = 10^3^, and *k*_6_ = 10^3^, and (d) α* = 1.0, *k*_5_ = 10^4^, and *k*_6_ = 10^3^. The following set of parameters was used: *D*_A_ = *D*_B_ = 0.1, *D*_C_ = 0.01, c* = 0.05, p* = 2.0, Δ*x* = 10^–3^ (grid spacing), and Δ*t* = 5 × 10^–7^ (time step).

The initial step in the precipitation mechanism is the formation of the intermediate shown in eq 2, and a sufficiently large reaction rate constant (*k*_1_) means that it is readily formed. When the concentration of the intermediate exceeds the critical threshold concentration (c*), the intermediates aggregate each other and subsequently transformed to crystalline precipitates described in eq 3. Once the precipitate is generated, it can grow easily by absorbing intermediates or constituent ions into them in the threshold-limited processes, as shown in eqs 4 and 5. In our previous report, eq 3 was an essential step to simulate the asymmetric microstructure of a single band, and *k*_2_ is much smaller than other reaction rate constants, which means that the aggregation of intermediates is a rate-determining step and that the overall precipitation patterns mainly result from eq 3.^21^ In this work, the Liesegang pattern varied by a function of PAA additives was successfully simulated by applying a threshold concentration (α*) in eq 4 and introducing a reversible step describing the interaction between Ca^2+^ and PAA (eqs 6 and 7) (Figure 5). The reaction rate constant of the formation of Ca^2+^-PAA complex (*k*_5_) is increased as the PAA content increased. The width of the precipitation bands decreased as the value of the threshold concentration became higher (Figure 5b, c). In the excessive threshold condition, the periodic pattern disappeared and only a continuous band was formed, which is similar to the experimental result (Figure 5d).

**Figure 6:**
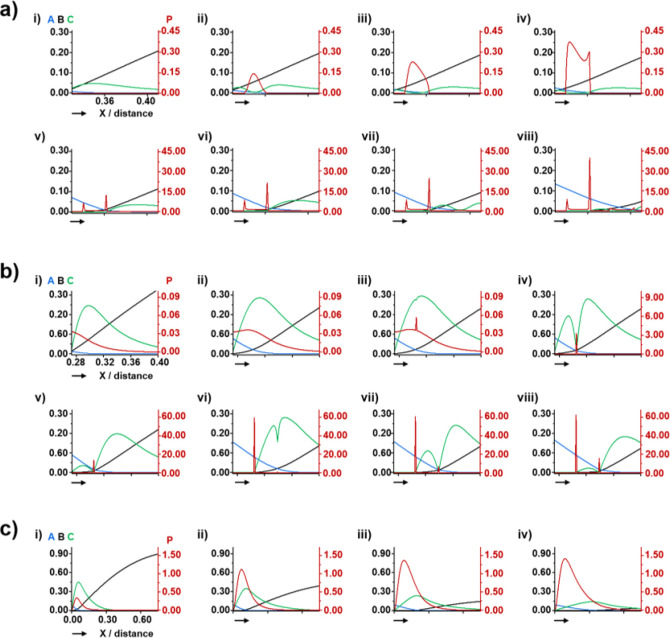
Simulated concentration of A (Ca^2+^; blue line), B (HPO_4_^2–^; black line), C (intermediate; green line), and P (precipitate; red line) when the threshold in eq 4 is (a) α* = 0, *k*_5_ = 0, and *k*_6_ = 0, (b) α* = 0.1, *k*_5_ = 10^3^, and *k*_6_ = 10^3^, and (c) α* = 1.0, *k*_5_ = 10^4^, and *k*_6_ = 10^3^. The band development as a function of the dimensionless time at (a) (i) 1.1600, (ii) 1.1900, (iii) 1.2100, (iv) 1.2400, (v) 1.4100, (vi) 1.4600, (vii) 1.4800, (viii) 1.6300, (b) (i) 0.9300, (ii) 1.1400, (iii) 1.1494, (iv) 1.1534, (v) 1.1800, (vi) 1.4200, (vii) 1.4300, (viii) 1.4600 and (c) (i) 1.0000, (ii) 4.0000, (iii) 7.0000, (iv) 10.0000. The left *y*-axis (black) is the concentrations of A, B, and C, and the right *y*-axis (red) is that of P. The following set of parameters was used: *D*_A_ = *D*_B_ = 0.1, *D*_C_ = 0.01, c* = 0.05, p* = 2.0, Δ*x* = 10^–3^ (grid spacing), and Δ*t* = 5 × 10^–7^ (time step).

The detailed process of band formation was analyzed based on the numerical simulations (Figure 6). In a control simulation, asymmetric bands were formed as mentioned in our previous work (Figure 6a).^21^ Precipitation started at the middle of the *n*th band while consuming neighboring intermediates which subsequently decreased its concentration. Because Ca^2+^ was incessantly supplied from its reservoir, the precipitate was first formed in the region nearer to the reservoir than to the central point, which resulted in the formation of the spike. Then, the central point was covered with some precipitates, and the spire began to develop because the intermediates only exist at the end of the band. Because the concentration of the intermediates (green line) decreased in the first band as a result of their aggregation and attachment to the precipitate, the intermediates presented in front of a reaction front are gathered by backward diffusion because of the concentration gradient. Finally, more crystals were precipitated at the end of the band, leading to the further development of the spire structure. The depletion caused by the backward mass transport of intermediates resulted in the void regions, in which the concentration of intermediates did not exceed the critical threshold. In the presence of threshold in the C + P step, the concentration of the intermediates could increase beyond the threshold only at a specific position, which resulted in precipitation in a narrow region (Figure 6b). It is noticeable that the band was abruptly formed as soon as the concentration of intermediates exceeded the threshold (Figure 6b, ii–iv). The simulation nicely matches with an experimental result; the narrow band was quickly developed in the presence of 0.1 and 1 mg/mL PAA, while the wide band with an asymmetric microstructure forms slowly in a control experiment (Figure 2a). The narrow band was grown by gathering the intermediates in both sides of the band, but it is mostly attributed to intermediates left behind the reaction front rather than those in the front of the band.^47,48^ The accumulated intermediates in front of the band were used for the growth of the next band (Figure 6b, vi–viii). This simulation result indicates that the narrow band was mainly developed by the forward mass transport of the intermediates accumulated behind the reaction front. In the case of high PAA concentration, the precipitation reaction was strongly inhibited by an excessively high threshold (Figure 6c). Thus, a tiny amount of precipitate was continuously and slowly deposited in the whole region without producing local accumulation of intermediates during the band formation.

### Mechanisms

**Figure 7:**
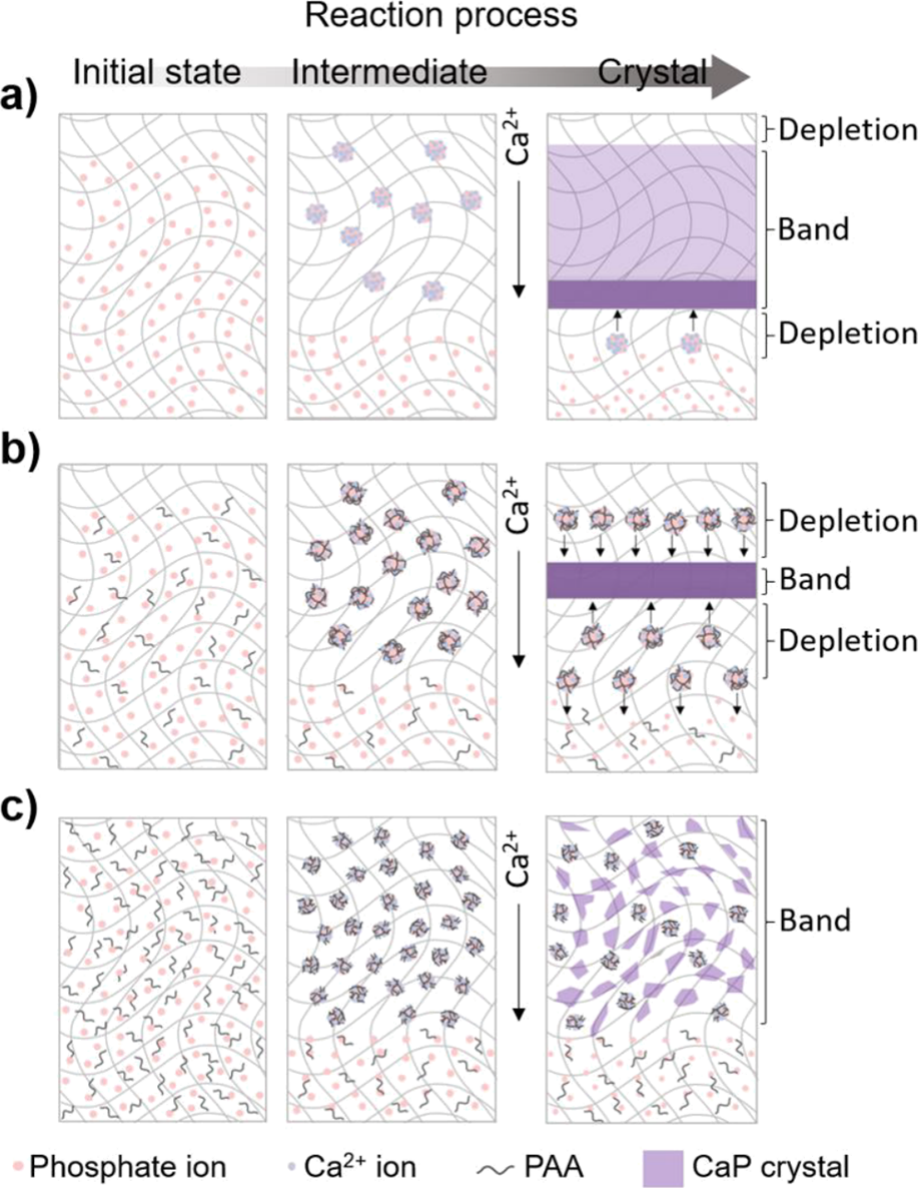
Scheme of band formation with (a) no PAA (control), (b) low PAA concentration, and (c) high PAA concentration.

Based on the inhibition on the CaP precipitate growth by PAA, the mechanism of each CaP band formation on different PAA concentrations was proposed on a microscopic scale (Figure 6). In our simulation, crystallization occurred through formation (eq 2), aggregation (eq 3), and attachment (eq 4) of the intermediate, as well as the attachment of ions (eq 5). It should be noted that the effect of PAA concentration on the pattern formation was successfully simulated by applying a critical threshold in eq 4, which implies that the variation of the patterns mainly resulted from retardation in the attachment of the intermediate to the precipitate.^22,49^ Therefore, the addition of PAA changed the reactivity and stability of amorphous intermediates, which finally resulted in a variation in precipitation patterns. Previous studies have demonstrated that soluble organic PAA containing COO^–^ can stabilize the amorphous precursors of CaP by sequestering calcium and phosphate ions, which results in the delay of the crystal growth and precipitation.^34^ In our system, the intermediates are interpreted as amorphous precursors, and the concentration of PAA can modulate the stability of the precursor, which is related to the critical threshold shown in eq 4. Because the factors for the inhibition of nucleation and crystal growth are completely excluded in the control, the mineralization is readily initiated at the relatively low level of supersaturation, leading to the expansion of the nucleation region and formation of wide bands (Figure 7a). As the stability of the precursor decreased, the precipitate easily grow by the particle attachment of intermediates (eq 4), which resulted in a larger size of crystals compared with those formed with PAA. The average size and crystallinity of OCP in a control (in the absence of PAA) and a sample with 0.01 mg/mL PAA were also determined using full width at half maximum (FWHM) of the 4.7° peak, the main peak of OCP. The FWHM value of the control (0.011327 radian) was almost identical compared with that of the 0.01 mg/mL PAA sample (0.010705 radian), which indicates that the crystallinity of OCP was not altered by the presence of PAA. Furthermore, the size of the crystallite was also calculated to be similar in control (12.25 nm) and 0.01 mg/mL PAA conditions (12.96 nm) according to Scherrer’s equation. In fact, the peak broadening in XRD is sensitive to the single crystalline domain, and the result of calculation with Scherrer’s equation was not the total size of aggregates but the size of the individual single domain.^50^ This suggests that the final crystalline precipitates consist of several smaller crystalline domains that have a similar size. Considering that the growth of precipitates occurred mainly by the aggregation of intermediates based on the simulation, aggregated precursors subsequently transformed into crystalline precipitates that have similar crystallinity without merging with each other. However, the aggregation of intermediates is inhibited in the presence of PAA, which diminishes the total size of final aggregates. Notably, the backward diffusion of the precursor formed a spire and produced the depleted zone in the last stage of band formation (Figure 7a).

In the presence of PAA in a hydrogel, the PAA adsorbed on the surface of ACP-blocked incoming constituent ions and increased the stability of the precursors.^36,38^ Thus, the level of threshold should be increased for crystallization. In the presence of a low PAA concentration, the concentration of precursors surpassed the crystallization threshold only in the limited point at the center of the band (Figure 7b). As soon as the nucleation started after a long period of incubation, stabilized precursors were rapidly dragged to the nucleation point from both sides, which resulted in narrow bands. Although the precursors were supplied from both sides of the nucleation point, those left behind the nucleation point were more contributed to the growth of the band through forward diffusion. Because the overall crystallization was retarded by stabilizing the precursors, the total size of the formed aggregated crystals was reduced by mainly inhibiting the attachment of precursors on the surface of the crystal, although the size of the single domain was maintained. In addition, once the precursor transformed into the crystalline precipitate, its crystallinity was not different from the precipitates formed without PAA.

In the presence of a great amount of PAA, the total density of precipitation is remarkably reduced because of an extremely elevated threshold for crystal growth. As a result, the precipitation was continuously formed in an extremely low density because there was no abrupt nucleation which induced a depleted zone for periodic patterns (Figure 7c). Instead, tiny precipitates were adsorbed on the hydrogel network.

## Conclusions

In this study, we demonstrated a bioinspired method to control and engineer the precipitation pattern of Liesegang bands by adding an organic polymer (PAA) that stabilized intermediates in the gelatin hydrogel. In previous studies, PAA was successfully used in the synthesis of amorphous calcium carbonate spheres and quantum dots^51,52^ and crystallization control of barium sulfate.^53^ In these studies, PAA affected the microscopic properties of the formed crystals. However, here, we showed that PAA can affect the macroscopic morphology of the pattern consisting of those small crystals. The width of the formed band drastically decreased in the presence of PAA. However, at high PAA concentrations, continuous precipitation was observed. While ACP was commonly observed at the early mineralization of CaP under all experimental conditions, OCP was the main crystalline phase of CaP after 5 days of reaction in the control experiment, and the addition of PAA favored the formation of ACP, which is considered the precursor phase of OCP. It was found that the overall size of precipitates was diminished as the concentration of PAA increased. These results indicate that increasing the amount of PAA stabilizes the transient ACP by retarding the transformation of ACP to OCP and hinders the overall crystallization, including nucleation and growth. To explain our experimental observation, we modified our previous reaction–diffusion model by varying the threshold concentrations in the attachment of intermediate processes. Based on simulation results, we suggest that the amorphous precursor of CaP was stabilized by the interaction between PAA and ACP that inhibited the growth and precipitation of CaP. This stabilization effect of PAA on the precursor induces different precipitation patterns depending on the PAA concentration. Previously, we found that the crystallization was initialized from the central part of the band because of the crossed initial concentration gradient of Ca^2+^ and HPO_4_^2–^ in the one-dimensional (1D) diffusion system.^21^ In the moderate PAA concentration system, the level of supersaturation to surpass the threshold was increased by PAA. Thus, nucleation can occur in the limited area near the center of the band, resulting in a diminished bandwidth of the Liesegang band. Notably, in the course of band formation, the spire was developed by the backward diffusion of precursors, producing the depleted zone in a control, but the growth of the narrow band resulted from the diffusion of the precursor phase from both sides. At a high PAA concentration, because the crystallization of CaP was highly suppressed, the local decrease in the concentration of precursors was too small to induce backward diffusion to form periodic patterns. Therefore, a continuous band was formed in the high PAA experiment.

## Supporting Information Available

The following file is available free of charge. Spacing, time, and width laws of the Liesegang pattern in non-PAA, 0.1 and 1 mg/mL of PAA, pH change of hydrogels as a function of distance after 5 days, XRD analysis of samples crystallized for 1 h in non-PAA, 0.1, 1, and 2 mg/mL of PAA conditions, and a magnified SEM micrograph of non-PAA, 0.01, 1.0 and 2.0 mg/mL of PAA conditions and the native gelatin hydrogel (PDF)

## Abbreviations

CaP: calcium phosphate
OCP: octacalcium phosphate
ACP: amorphous calcium phosphate
PAA: poly(acrylic acid)
PILP: polymer-induced liquid precursor
