# Supplementary material for: Bioinspired Control of Calcium Phosphate Liesegang Patterns Using Anionic Polyelectrolytes
Source: Langmuir. 2022 Feb 11;38(8):2515–24. doi: 10.1021/acs.langmuir.1c02980 (PMC8892956; doi:10.1021/acs.langmuir.1c02980)
Supplement: Supplementary file 1 — la1c02980_si_001.pdf [file la1c02980_si_001.pdf]

Supporting Information

Bio-inspired Control of Calcium Phosphate Liesegang Patterns Using Anionic Polyelectrolytes

Young Shin Cho[a,§], Miyoung Moon[a,§], Gábor Holló[b], István Lagzi[,b,c], and Sung Ho Yang[*,a]*

[a]Department of Chemistry Education, Korea National University of Education (KNUE), Chungbuk, 28173, Republic of Korea

[b]MTA-BME Condensed Matter Physics Research Group, Budapest University of Technology and Economics, Budapest, Hungary

[c]Department of Physics, Budapest University of Technology and Economics, Budapest, Hungary

*E-mail: sunghoyang@knue.ac.kr, lagzi.istvan.laszlo@ttk.bme.hu

[§]*Y.S.C. and M.M. contributed equally to this paper*

Table of Contents:

Figure S1. Spacing, time, and width laws of Liesegang pattern in non-PAA, 0.1 and 1 mg/ml of PAA.

Figure S2. The pH change of hydrogels as a function of distance after 5 days.

Figure S3. XRD analysis of non PAA (control) (black), 0.01 (red), 1.0 (blue) and 2.0 mg/mL (green) of PAA. Samples were crystallized for 1 h.

Figure S4. A magnified SEM micrograph of (a) non PAA (control), (b) 0.01, (c) 1.0 and (d) 2.0 mg/mL PAA and (e) native gelatin hydrogel.

.

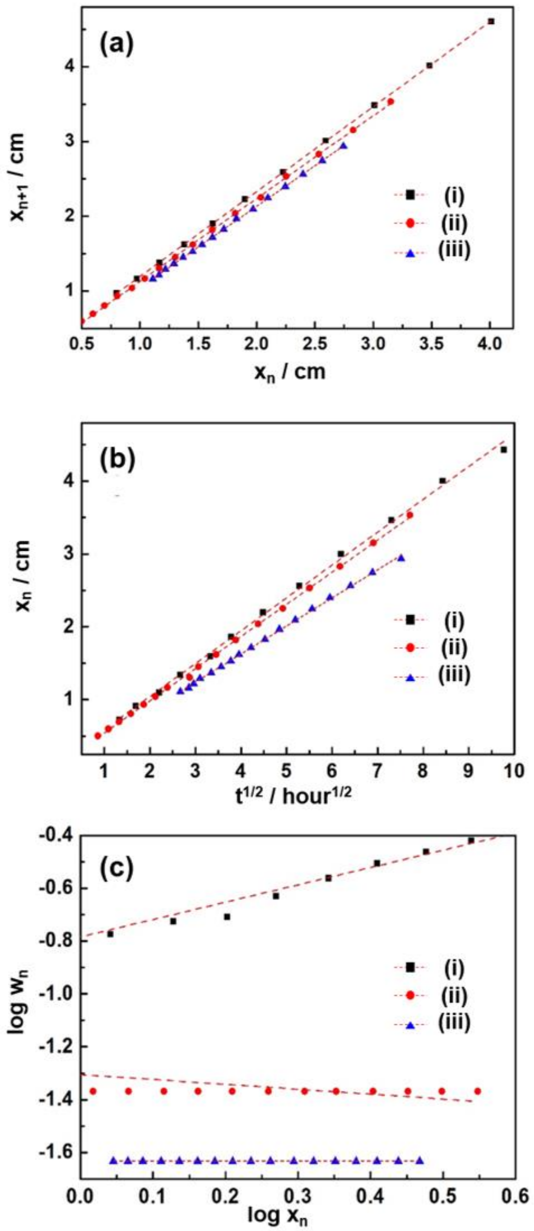

Figure S1. (a) Spacing, (b) time, and (c) width laws of Liesegang pattern in (i) non PAA, (ii) 0.01 and (iii) 1 mg/mL of PAA. The quantities presented in the graphs are x_n, t_n, and w_n the distance of the *n*th band measured from the liquid-gel interface, the formation time, and the thickness of the of the *n*th band, respectively. Liesegang patterns were confirmed by three law: spacing law ($p = (x_{n+1} - x_n)/(x_n)$, the ratio ($p$) of the positions of the consecutive bands measured from gel surface (x_{n+1} and x_n) is constant), time law ($x_n = q_1\sqrt{t_n}$, the position of *n*th band (x_n) is linearly proportional to the square route of its formation time (t_n)), and width law ($w_n = q_2 {x_n}^a$, the widths of bands (w_n) grow with their position (x_n))

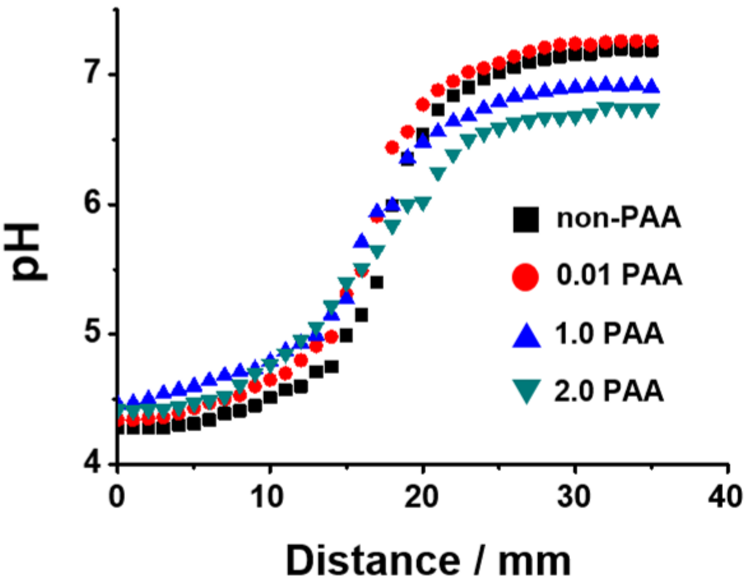

Figure S2. The pH change of hydrogels as a function of distance after 5 days.

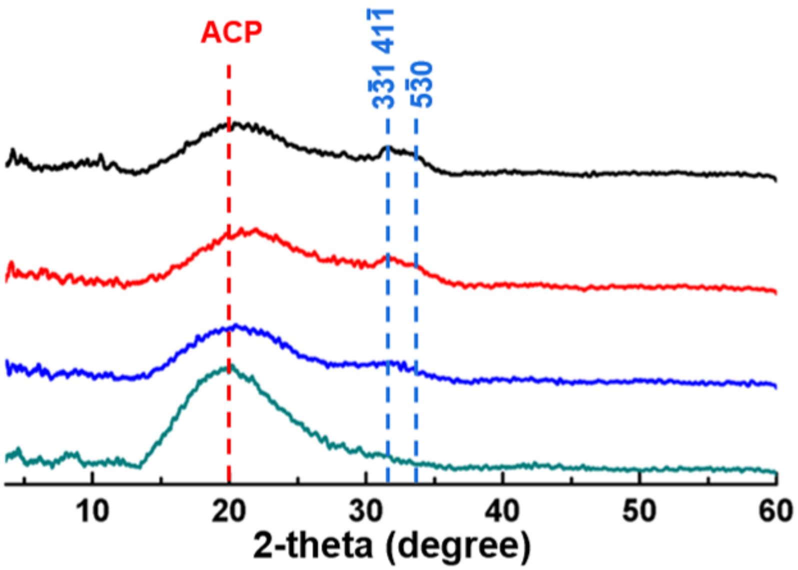

Figure S3. XRD of non PAA (control) (black), 0.01 (red), 1.0 (blue) and 2.0 mg/mL (green) of PAA. Samples were crystallized for 1 h. The red dashed line shows the characteristic broad peak at ~20° indicating the presence of ACP. The blue dashed line represents the small peaks assigned to (3-31)/(41-1) and (5-30) of OCP.

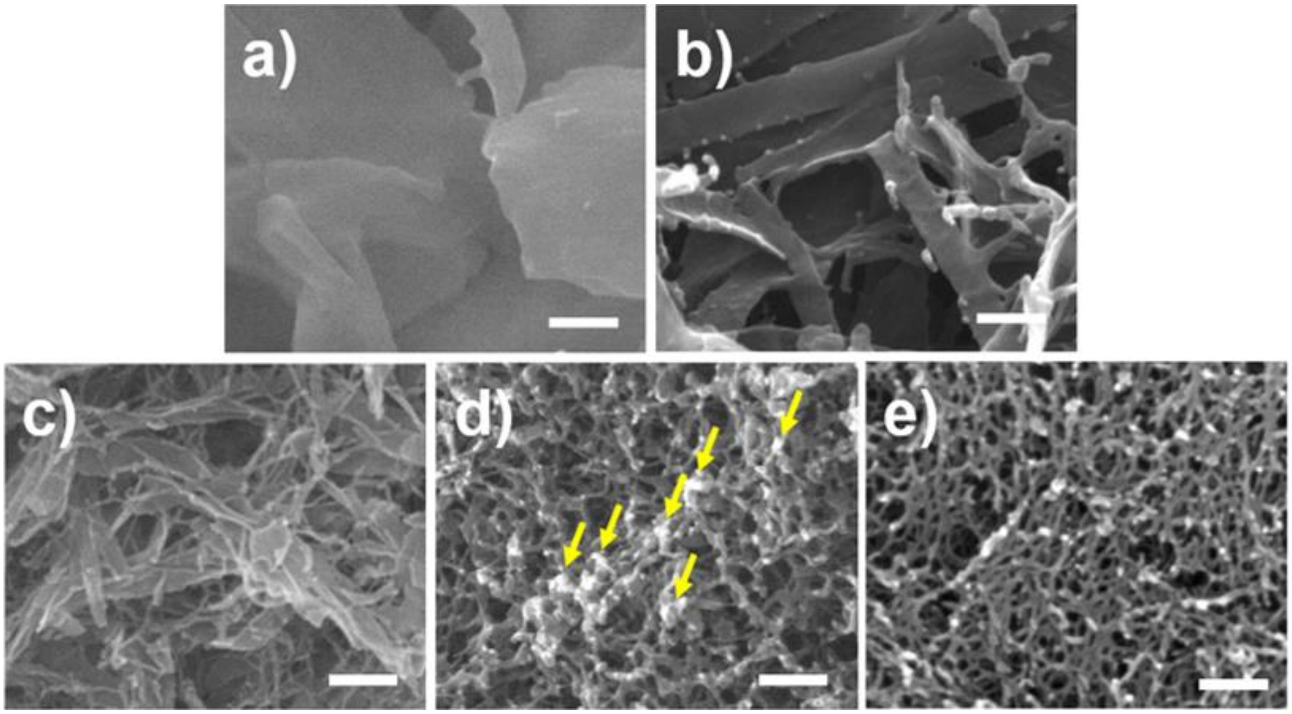

Figure S4. Magnified SEM micrographs of CaP crystals in a hydrogel formed with a) 0, b) 0.01, c) 1.0, d) 2.0 mg/mL of PAA, and e) native gelatin hydrogel. All SEM micrographs were obtained by observing cross sections of samples. As the amount of PAA added increases, the size of the crystals becomes smaller and the crystals appear adsorbed form on the gel network. The scale bars are 200 nm. Yellow arrows indicate dangled CaP particles on gelatin hydrogel.
